# BENS−B5G: Blockchain-Enabled Network Slicing in 5G and Beyond-5G (B5G) Networks

**DOI:** 10.3390/s22166068

**Published:** 2022-08-14

**Authors:** Saurabh Singh, C. Rajesh Babu, Kadiyala Ramana, In-Ho Ra, Byungun Yoon

**Affiliations:** 1 Department of Industrial and System Engineering, Dongguk University, Seoul 04620, Korea; 2 Department of Networking and Communications, SRM Institute of Science and Technology, Chennai 603203, India; 3 Department of IT, Chaitanya Bharathi Institute Technology, Hyderabad 500075, India; 4 School of Computer, Information and Communication Engineering, Kunsan National University, Gunsan 54150, Korea

**Keywords:** blockchain, network slicing, 5G communications, radio access network, 5G-CORE network functions, beyond-5G

## Abstract

Fifth-generation (5G) technology is anticipated to allow a slew of novel applications across a variety of industries. The wireless communication of the 5G and Beyond-5G (B5G) networks will accommodate a wide variety of services and user expectations, including intense end-user connectivity, sub-1 ms delay, and a transmission rate of 100 Gbps. Network slicing is envisioned as an appropriate technique that can meet these disparate requirements. The intrinsic qualities of a blockchain, which has lately acquired prominence, mean that it is critical for the 5G network and B5G networks. In particular, the incorporation of blockchain technology into B5G enables the network to effectively monitor and control resource utilization and sharing. Using blockchain technology, a network-slicing architecture referred to as the Blockchain Consensus Framework is introduced that allows resource providers to dynamically contract resources, especially the radio access network (RAN) schedule, to guarantee that their end-to-end services are effortlessly executed. The core of our methodology is comprehensive service procurement, which offers the fine-grained adaptive allocation of resources through a blockchain-based consensus mechanism. Our objective is to have Primary User—Secondary User (PU—SU) interactions with a variety of services, while minimizing the operation and maintenance costs of the 5G service providers. A Blockchain-Enabled Network Slicing Model (BENS), which is a learning-based algorithm, is incorporated to handle the spectrum resource allocation in a sophisticate manner. The performance and inferences of the proposed work are analyzed in detail.

## 1. Introduction

As a result of the Internet of Things era, new time- and mission-critical applications that incorporate 5G or B5G have been created for every sector of human activity. These end-to-end applications are organized using a series of network services [1]. As a result, infrastructure operators must bring computational capabilities closer to end-users to meet the delay requirements of cloud computing. Wireless data traffic will surge in the coming years as the number of mobile users and the wide range of bandwidth-hungry apps they use increase dramatically. Next-generation (5G) and 5G wireless communication networks will support a broader communication ecosystem, including the Internet of Things (IoT) and Internet of Vehicles (IoV) [2]. The 5G and beyond 5G wireless communications are expected to constitute the foundation for several new applications to enable this progress. Customer’s needs for applications have a wide range of complexity and customers will want to be met with 5G and beyond wireless communications. Some vertical sectors, such as industrial automation control systems and the Internet of Vehicles, need an exceptionally high reliability and low latency communications to meet rigorous QoS requirements [3]. To meet the diverse and personalized QoS needs of 5G and beyond networks, it is necessary to re-examine networking technology and network design. The International Telecommunication Union (ITU) has categorized three sorts of users in terms of service classification: enhanced mobile broadband (eMBB), ultra-reliable low-latency communications (URLLC), and massive machine-type communications (mMTC), which are all examples of new technologies that are being developed to meet the needs of today’s mobile devices [3]. Enterprises are searching for creative solutions to satisfy their demands and address new prospects as a result of the arrival of new technologies brought about by 5G, as well as the new business chances that have been created across all sectors. When it comes to enterprise customers, they demand automated business and operational processes from the time they buy the service through activation, delivery, and decommissioning. They anticipate that services will be provided more quickly while maintaining a high level of safety. Through the use of network slicing, communication service providers can fulfil all of the requirements posed by their corporate clients.

**Figure 1:**
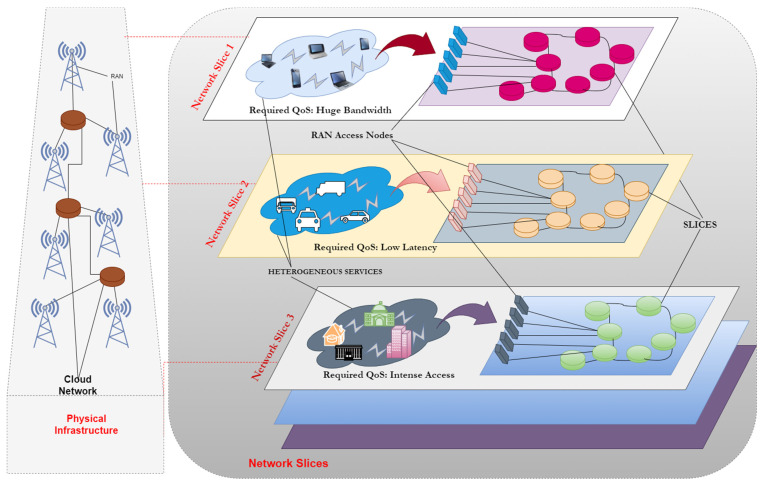
Network slicing scenario.

The 5G network design, based on network slicing is expected to play a significant role in the future generation of networks. The virtual network is described as a slice in network slicing, which allows numerous independent and separated virtual networks to coexist in the same physical network infrastructure. Using Software Defined Networks (SDN) and Network Function Virtualization (NFV), networks may be sliced to accommodate new services with a broad range of needs, while still using the same physical network (PN). Slices are created by abstracting control logic and resources from the SDN controller and making them available to the SDN nodes. SDN network slicing also makes it possible for several tenants to share the same PN resources. On the other hand, NFV was created to address a lack of particular communication equipment in the market. Virtual network functions are at the heart of NFV, and they may be performed on standard servers without the need for specialized hardware. Due to these attractive benefits, network slicing is often used [4]. Multi-tenancy is supported via network slicing, which allows the same physical infrastructure to be used by many virtual network operators. Network slicing may be used to provide differentiated service and meet service level agreements. Network slicing facilitates the capacity to build and alter network slices on demand, which increases the adaptability and flexibility of network administration. Figure 1 shows a network situation using network slicing. To simplify management and coordination, network slices are used to represent the underlying physical infrastructure. Core networks (CNs), radio access networks (RANs), and other physical resources are separated into many logical components, resulting in various network slices that may be tailored to meet the needs of different users [5]. Network slicing has numerous slices for diverse services and sits above the underlying layer of the network stack. It is widely regarded as one of the most promising technologies for 5G and beyond networks because of its ability to provide a wide range of QoS requirements for diverse services.

By splitting the same PN into many isolated logical networks, RAN slicing may deliver tailored services for isolated logical networks. This cost-effective and high-efficiency network management approach is built on the concept of PN sharing. Ref. [6] has found that RAN sharing might save the global economy about 60 billion dollars by 2022 in terms of both capital and operating expenses. The 3GPP has conducted an extensive study of 5G network slicing in practice.

To meet diverse QoS requirements, it is necessary to find a method for allocating network resources that is both flexible and efficient, and this is where RAN slicing comes into play. RAN slicing allows network operators to effectively and flexibly distribute resources based on the performance needs of individual users. In addition to wireless resources, these resources include those for computing and cache storage. The resources may be used more efficiently and at a higher rate with the help of resource-allocation technology, which can also merge diverse slices. Network slicing may be categorized as either a static or dynamic allocation, depending on the circumstance. After deciding on a resource allocation and mapping approach for network slicing, the allocation will remain static, no matter what changes occur in the environment. A key characteristic of dynamic resource management is the capacity to adapt resource-allocation tactics in response to changes in the environment, ultimately resulting in improved communication service quality.

It is critical to construct cross-domain RAN slicing that incorporates different operators and infrastructure suppliers. For example, an autonomous driving module requires a RAN slice that can cover a large geographical region by using services from a variety of local operators located across the city [7]. On the other hand, traditional cross-domain orchestrators are built on a master–slave design, which has a number of problems. A self-interested master virtual mobile network operator (VMNO) can capture super profits over other players, preventing them from entering the system, since they are responsible for collecting occupants’ slice requests, resource allocations, and incentive distributions. Second, the master VMNO must negotiate the cost of access to all the tenants’ resources on a frequently time-intensive and wasteful basis. To alleviate incumbents’ concerns about the ‘Master’, both research and commerce have recently laid great emphasis on the blockchain, a distributed immutable data recorder that is capable of establishing trust between untrusted peers. Additionally, smart contracts, which are contracts that have been encoded and managed by computers, are optionally utilized by the blockchain without the approval of the authority. With smart contracts, blockchain technology enables the management systems to easily handle the complicated operations.

In this paper, we present a *tabula rasa* model referred to as BeNS−B5G, a RAN slicing framework based on blockchain, which improves fairness and orchestration efficiency, and we use reinforcement learning methods to further improve its performance. In contrast to conventional designs, the suggested one is distributed, with several cloud service providers (CSPs), virtual machine network operators (VMNOs), and infrastructure providers (InPs) co-managing a blockchain system. Rather than merely listening to a committed master, all incumbents fight for leadership. Without the need for human discussions, smart contracts running on the blockchain automatically record resource statuses, process RAN slice requests, and pay incentives to resource providers. When the tenants release the slice requests, an intelligent resource optimizer (RO) is included to assist VMNOs, in determining the optimal resource combination that meets the requested QoS criteria of RAN slice.

The rest of this article is organized in the following manner. We begin with a comprehensive analysis of RAN slicing and the blockchain method, followed by a discussion of the obstacles associated with implementing the blockchain-enabled network slicing framework. We propose a RAN slicing architecture that leverages the distributed and automated nature of blockchain technology and incorporates a novel consensus mechanism to assure system performance, while aligning with incumbents’ economic objectives. Following that, we further reduce slice requesters’ costs by using Federated Learning (FL) methods to determine the ideal resource combination. The learning algorithm’s benefits are assessed via simulations, and associated future approaches are considered as well as outstanding concerns.

## 2. Related Work

The exhaustive related work on cognitive radio networks has been studied based on the following categories:

- Cognitive Radio over 5G and Beyond Networks;
- Network Management in 5G and Beyond Networks.

### 2.1. Cognitive Radio over 5G and beyond Networks

Abubakar Makarf et al. (2020) [8] explored combining the Radio Information System (RIS) and Monte Carlo concept within a network to maximize the potential benefits. Two different RIS-based network models were investigated, and many performance measures connected with the CR secondary user were implemented. The obtained equations were validated using Monte Carlo simulations. In the presence of an RIS-enhanced main network, the results showed the influence of key system parameters and a clear improvement in the CR network. According to Zhaoyuan Shi et al., the massive MIMO which is, underlying the cognitive radio user selection schema, is aware of the QoS requirements of the channel requested (2019) [9]. There are two major ways in which a CR may be implemented: The Channel State Indicator (CSI) of any cross-network is inaccessible at the secondary base station (SBS), but the SBS has access to the CSI of the cross-channel channel state. Low-complexity algorithms for increasing users while using the least amount of power (IUMP) and methods for decreasing users while using the most amount of power (DUMP) were developed to solve user selection via power allocation. The intractable challenge was addressed using a deep reinforcement-learning-based method, enabling the SBS to accomplish effective and intelligent user selection. In simulations, these algorithms dramatically outperform the current user selection approaches. Our neural network was able to rapidly learn the best user selection strategy in an unknown dynamic environment with a high success rate and fast convergence, as the findings also revealed. Kok-Lim Alvin Yau et al. [10] focused on how CR and the Cognition Cycle have been integrated into 5G to deliver spectrum efficiency, energy efficiency, enhanced quality of service and experience, and cost-efficiency (2018). Open research opportunities and platform implementation were made accessible to inspire new research in this area. Gianfranco Nencioni et al. (2018) [11] discussed the fifth-generation (5G) of cellular networks, which is expected to represent a significant advancement in wireless technology. A variety of new wireless technologies will be implemented to better serve 5G’s wide set of requirements, including upgrades to the radio access network. It was demonstrated that the convergence of many communication technologies has been facilitated by embedding softwarizations such as Software-Defined Networking (SDN) and Network Functions Virtualization (NFV). Through network slicing, 5G networks may be constructed at low cost. By using an SDN/NFV architecture, 5G radio access and core networks will be able to deliver network services more efficiently, flexibly, and in a more scalable manner. The authors also discussed software-defined 5G radio access and core networks, as well as a wide range of future research topics in orchestration and control. Johana Hernández et al. proposed cognitive radio management (2018) [12].

### 2.2. Network Management in 5G and beyond Networks

The most available route for the opportunistic transmission of secondary user data is chosen throughout the decision-making process as a result of the main user characterization-model’s efficiency. It was claimed that an approach based on deep learning and long short-term memory might lessen the forecasting error now present in future significant user estimates in the Global System for Mobile Communication (GSM) and WiFi frequency bands. When compared to alternative approaches, such as multi-layer perceptron neural networks, Bayesian networks, and adaptive neuro-fuzzy inference systems, the results indicate that a lengthy short-term memory may significantly enhance the estimates of channel utilization (ANFIS−Grid). The neural structure has input, forget, and output gates, which complicates its implementation in cognitive radio networks (CRNs) based on core network topologies, despite the fact that a long short-term memory fared better at time series forecasting. Aaron Yi Ding et al. developed the criteria for assessing the restrictions of 5G-driven applications (2018) [13]. The usual hurdles and needs for various application domains were studied using 5G networks as a basis. The major objective was to create a network architecture that could adapt to changing traffic patterns, while also supporting diverse technologies, such as edge computing, blockchain-based distributed ledgers, software-defined networking, and virtualization. We underlined the need to perform 5G application pilots to better understand how 5G networks are deployed and utilized in different vertical industries. Xingjian Li et al. investigated the issue of spectrum sharing in a cognitive radio system with a main and secondary user (2018) [14]. Secondary users are at odds with prime users. In particular, it was assumed that the primary user would alter its transmitted power in accordance with a pre-defined power management strategy. The secondary user has no idea what the main user’s transmission power and power control method are. For the secondary user, a power control system based on learning was developed to share the same spectrum with the main. To assist the secondary user, a network of sensor nodes was strategically placed across the wireless network to collect data on the received signal strength. The secondary user’s transmission power may be automatically adjusted using a deep reinforcement learning algorithm. This may be performed after a few rounds of interaction with the principal user. The results showed that secondary users might effectively connect with main users to achieve the desired state from any beginning situation in a few steps. When Maria Massaro et al. (2017) [15] examined the Licensed Shared Access (LSA) and Shared Access Spectrum (SAS) regimes in the EU, they discovered significant disparities between the LSA and SAS regimes. To acquire information on the technical and regulatory components of current and forthcoming spectrum-sharing regimes, policy documents, research publications, position papers, and analytical studies were studied. The LSA regime is notable for providing mobile operators with the regulatory certainty they need to invest in 5G, while also granting them access to an additional spectrum below 6 GHz. Other spectrum-sharing regimes will not safeguard cell operators from hazardous interference or ensure trustworthy Quality of Service (QoS) while utilizing sub-6 GHz airwaves. Due to its lower level of technical complexity, the LSA regime can be deployed more rapidly and with less effort when the two techniques are compared. However, as technology progresses, the LSA regime is projected to be surpassed in the long run by the SAS regime. More persons may share the same frequency channels under the SAS setup. A cognitive WLAN overlay over an OFDMA TDD main network was tested for saturation throughput by Parisa Rahimzadeh et al. (2017) [16], e.g., using LTE or WiMAX. The successful node delivers its data packet in the main network’s downlink and uplink subframes that have empty resource blocks (RBs). Unlike the OFDMA structure and time-scheduled resources in the primary network, the opportunity length in the secondary network does not follow a straightforward exponential on–off pattern. There is a mathematical model for the dynamic behavior of secondary node opportunities and contentions that incorporates a discrete-time Markov chain and two connected open multi-class queueing networks (QNs). As a random number is created when data are downloaded and uploaded, our research includes the random packet transmission time on WLANs, the dependency on the number of empty RBs in the subsequent frames, and aspects of the 802.11 MAC protocol. Multiple resource allotments were inserted into the main network for the purpose of conducting the analysis. We were able to demonstrate the correctness of our method via simulations in a variety of different situations. Cheng Wu et al. (2016) [17] developed a multi-agent reinforcement learning-based spectrum management approach. For efficient spectrum and transmit power allocation, the approach employed value functions to evaluate the advantages of different transmission features, maximizing long-term return. Using a variety of learning factors, students were exposed to a variety of real-world circumstances, and their communication skills were evaluated. A Kanerva-based function approximation was utilized to enhance the management of large CRNs, and to examine the impact on communication performance in these networks. The proposed reinforcement learning-based spectrum management in a cognitive radio ad hoc network is shown to considerably minimize interference to licensed users, while preserving a high probability of successful transmissions in this network. The secondary users’ average sum rate was increased by Yang Yang et al. (2017) [18] by determining the best way to access and regulate their power in multiple bands (ASR). We represented the random distributions of PUs and SUs using Poisson point processes (PPPs) based on stochastic geometry, from which we were able to compute the closed-form outage probability and estimate the ASR of SUs. On a number of bands, the ASR maximization problem included an outage probability. The optimal density of SUs with a given power was determined using closed-form convex optimization, and the optimal SU power was calculated and ASR convexity was checked. These findings prompted the creation of a spectrum access and power management strategy aimed at optimizing the ASR of SUs over several bands. The results of the simulations reveal that PUs and network interference limit SUs’ density and power, and that the proposed approach can achieve the maximum ASR for the SUs.

With the exhaustive survey on network management in 5G and beyond networks, it is becoming ever clearer that resource allocation becomes a tedious process with the dynamics of 5g and Beyond 5G networks. Hence, to focus on an efficient and reliable resource allocation technique, we incorporate a state-of-the-art blockchain-enabled network-slicing method with 5G and beyond networks.

## 3. Blockchain-Enabled Network Slicing in 5G

In this section, we develop a blockchain-enabled RAN slicing architecture that encompasses all stakeholders in the 5G and beyond network slicing scenario, ensuring invisible automation, transparency, and optimal interactions between them. The suggested design is consistent with the smart contract as a management platform paradigm, in which all services are supplied by contracts and are invoked by users through transactions. InPs, VMNOs, MNOs, and CSPs are all incumbent members of the RAN slicing architecture. All of these players are agents in the FL and blockchain-enabled RAN slicing, and all users except occupants are users [19]. The following criteria are used to choose agents. On the one hand, agents should have robust capabilities for storing and validating blockchain data, as well as the ability to remain online at all times. As a result, occupants would be less qualified for the task.

### 3.1. Spectrum Sharing and Slicing in 5G

We are proposing a dynamic spectrum sharing and slicing system using broad sensing to overcome this limitation of sharing most of the unused spectrum bands to gain additional spectrum resources for 5G.

Terminals and fixed sensors track the spectrum of use of a primary user in this system. Another system’s transmission spectrum or power must be dynamically identified from enormous tracking data when spectrum sharing is necessary. During spectrum sharing, the secondary user must determine whether or not they are interfering with the PU. Interference between the PU and SU is avoided by changing the transmission spectrum, power, or linked network. Regardless of whether the principal user is migrating or not, this technique may be used in a variety of systems. A paradigm for dynamic spectrum sharing in the 5G−CORE network is also proposed in this paper. We concentrate on the scenario in which the base station is responsible for implementing the dynamic spectrum-sharing features. As an addition to the DSA, we offer an implementation strategy for dynamic spectrum sharing.

**Figure 2:**
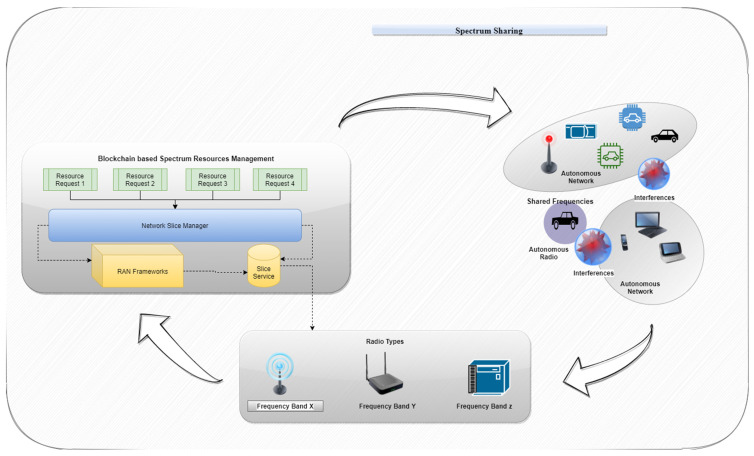
Dynamic spectrum-sharing environment.

Figure 2 shows the dynamic spectrum-sharing mechanism. Dynamic spectral monitoring, the sharing of existing spectral bands, and the coordination of the utilization of unused spectra comprise the system. Terminals and fixed sensors monitor the primary user’s spectrum utilization via dynamic spectrum monitoring. A spectrum sharing condition, such as the availability of a spectrum band or transmission power, is dynamically defined in the current spectral bands for sharing. During spectrum sharing, interference between primary and secondary users might alter the sharing conditions.

### 3.2. Bands That Share a Spectrum Range

The received powers of primary users with several terminals and fixed sensors are calculated using these functions. With terminals and mobile-dispersed monitoring, a watch can be kept on a large region. On the other hand, fixed sensors in shared spectrum bands can keep an eye on a wider spectral range. The base station receives a summary of the monitoring data. However, when the monitoring data is delivered, the system’s overhead would be enormous. Data collection through street-pass communication between terminals may be explored to reduce the quantity of transferred data. To determine how well the sharable spectrum is being utilized, and to avoid interference, data from the base station are collected and analyzed.

### 3.3. Dynamic Environmental Monitoring

This function determines whether large numbers of data should be shared. Fast and dynamic data analysis using edge computing and distributed databases are used to determine the current state of affairs. The interpolation and inference of frequent utilization situations are used to determine the sharing condition [20]. The secondary base station and secondary terminal interact according to the set conditions.

### 3.4. Utilization of Shared Spectrum Bands

PU—SU interference may arise while they switch locations throughout normal usage. To avoid interference, the secondary user’s transmission spectrum or power should be modified without affecting the PU. The PU—SU communication regions are estimated using the data gathered through monitoring. Autonomous radio coordination or autonomous network coordination is carried out based on the estimated regions [21]. The power or spectrum of the transmission in the same band may be changed automatically through radio coordination. If the secondary user is still interfering with the primary user after radio coordination, the network connection for the secondary user is replaced. Figure 2 shows the dynamic environmental changes of the PU and SU.

### 3.5. Dynamic Spectrum-Sharing Specification for the 5G−CORE Network

**Figure 3:**
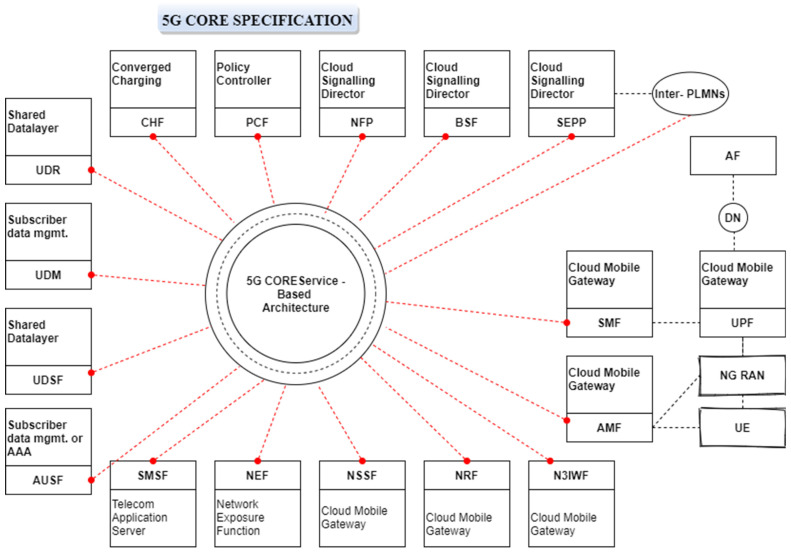
5G−CORE specification for dynamic spectrum sharing.

Figure 3 presents a framework for dynamic spectrum sharing in the 5G−CORE network, where three application situations in terms of the placement of the component functions are explored. Configuration the base station, on top of an AMF, and outside the 5G−CORE network are the first two possibilities.

#### 3.5.1. Scenario 1—Configuration in Radio Access Network

The Radio Access Network regulates the user equipment linked to the network. The RAN may use the sharable spectrum, and coordinate when interference occurs without a burden on the main network. The RAN cannot identify the interference until the primary user reaches the edge of the secondary user’s territory, since the RAN has information about its own area. This indicates the PU—SU interference. Therefore, the design becomes successful in communication systems where the primary user does not relocate.

#### 3.5.2. Scenario 2—Configuration of Access and Mobility Management Function

Since the core network may gather data from several RANs, it is possible to cover a large region by using multiple RANs. The core network is far more sensitive to the presence of the primary user than the RAN. As a result, in systems where the principal user travels, this use case is effective. There is a delay in detecting and coordinating interference, because the core network must gather data from many RANs.

The following section discusses the configuration of the RAN and a learning-based channel allocation mechanism for the specified model.

### 3.6. Configuration of Radio Access Network

The 5G cellular networks are divided into four sub-segments using a Multi-Layer Stratified Networking paradigm that incorporates Machine Learning and Cognitive Radio technology. We use a three-level MAS paradigm for the core network, secondary users, and primary users to create a complex Multi-Agent System that incorporates AI and Cognitive Radio technologies. In the age of AI, robots are able to see and learn from their surroundings in the same way as humans. Furthermore, in an Artificial Intelligence (AI) system, people may achieve great abilities via clustering algorithms. Meanwhile, a plethora of AI solutions have opened the way for optimizing network connectivity and allocating resources. Furthermore, 5G cellular networks may benefit from the merging of AI and CRNs, providing the network with the same intelligence and autonomy as a human. The AI and machine learning methods are presented in such a way that how far it is important to incorporate a learning-based system for the CRNs [22]. Reinforcement Learning can be collaborated with the CRNs for channel sensing and allocation and dynamic routing. With the help of RL, CRNs’ primary and secondary users may more efficiently use their local operating environments’ spectrum resources by using online learning behavior and adopting the best actions possible.

Our architecture integrates AI technologies with channel resource management and Base Stations’ resources management in 5G communications to ensure the QoS needs of CRN users, maximize spectrum utilization, and optimize the BSs resources control approach. While the reinforcement learning algorithm performs well in CRNs, learning strategies also rely on the network’s system features, which include solitary, multi-agent, hierarchical, and distributed networks. We have utilized multi-agent reinforcement learning for cooperative power distribution in CRNs. It is also claimed that CRNs use a reinforcement learning system to estimate the throughput and discover accessible idle channels. In addition, the distributed optimization technology of a heterogeneous small cell network is given. Cognitive Radio consumers may expect improved resource efficiency and QoS assurances with AI-based hierarchical and distributed network technologies.

## 4. Learning Based Slicing (Channel Resource Allocation) for the RAN in 5G-CORE

We examine the channel resource allocation technique for a Multi-Agent System with several primary users and secondary users. An agent is a CR user, and the environment considers all the PU—SUs over the 5G network. Each agent acquires information and makes decisions in response to an environmental input and spectrum resources are allocated in a dynamic manner to maximize each agent’s advantage. The Multi-Agent Reinforcement Learning method is seen in this light as addressing a decision problem in the Multi-Agent System. Additionally, we must investigate agent behavior, and provide an appropriate technique for Multi-Agent Reinforcement Learning [23]. From this vantage point, interventions need three components: an actor, a context, and rules. Each of these components is discussed in depth in the following sections. In the suggested paradigm, agents are basic, consisting of the PU and SU. Each agent is a self-contained entity capable of sensing, observation, learning, and decision making. These agents behave according to well-defined rules, while interacting with the environment of a CRN. We abstract a model of a Multi-Agent System that incorporates an intelligent Base Station control mechanism and dynamic spectrum allocation. Every agent decides on an action by observing, learning, and deriving a perceptive state from the environment

### 4.1. Environment

A partially observable Markov decision-making process can be used to define the environment. In the Multi-Agent System model, while an agent makes a decision, the rest of the agents become idle, and a Markov decision process is used to define the environment. While a PU-agent and environment interaction takes place, CRNs consider all other agents, including the PU and SU, to be the environmental components.

### 4.2. Rules

Rules are critical in Multi-Agent Reinforcement Learning. This article discusses two distinct kinds of agent rules: isomorphic or heterogeneous. Primary User-to-Primary User and Secondary User-to-Secondary User policies are isomorphic, while Primary User-to-Secondary User policies are heterogeneous agent rules. PUs–SUs interactions provide incentives and rules.

In the Multi-Agent System, Primary User-to-Primary User competition occurs. The Primary Users begin by acquiring channel resources given by BSs. The Primary Users then use a part of their resources and distribute the remnants to the Secondary Users. The Secondary Users must compete for unused channel resources. As a consequence, a system for resource allocation among Primary Users should be developed to ensure that their advantages are maximized.

### 4.3. Secondary User to Secondary User

The SU agent is the first to receive information about the channel’s resource consumption status, which includes the active channel count and the PU—SU count. Between the CR users, there are two sorts of relationships: competitive and neutral. Mathematically, we may assume that the numbers of channel resources, Primary Users, and Secondary Users are A, B, and C. Secondary Users are in a state of neutrality when A–B.C. As a consequence of bargaining among the Secondary Users, the different idle channel resources may be occupied. When A–B.C occurs, a competitive link between the Secondary Users exists as well as A–B subchannels. These Secondary Users compete directly for spectrum resources in the A–B subchannels. As a consequence, each Secondary User must develop an appropriate policy.

### 4.4. Primary Users to Secondary Users

PUs−SUs communicate and learn inside CRNs. PUs make the most efficient use of the spectrum resources made available by the Base Stations during the encounter. Following that, the Secondary Users may be allocated idle resources. As previously mentioned, N Secondary Users compete for spectrum resources with S = (s1, s2, …, sn) data needs. Primary and Secondary Users both strive to maximize resource usage in a dynamic multi-agent environment. To perform this, Primary Users must be able to distinguish Secondary Users and their data needs. Each Primary User has an allocation, a request, and a wait strategy. When a Primary User’s idle resources are adequate for provision to the Secondary Users, the Primary User chooses to wait; however, when the Primary User’s idle resources are insufficient for allocation to the Secondary Users, the Primary User decides to seek resources from other Primary Users. Thirdly, when another Primary User submits a request, the current Primary User determines whether to approve it.

### 4.5. Evaluation Methods

Machine learning is concerned with deriving a function from a noisy set of data, referred to as the training set, which was created by an unknown true function. Supervised learning and reinforcement learning are two machine learning techniques that are useful in this context. Supervised learning derives a function from a series of data pairs supplied by a supervisor, each of which has an input and a desired outcome. Artificial Neural Networks are a family of function approximators that may be customized for a particular job by adjusting their weights appropriately [24]. Training an ANN entails progressively adjusting its weights to minimize the error function between the ANN-represented function and the real function’s actual noisy data samples. The term “backpropagation” refers to the practice of applying gradients to ANNs. On the other hand, reinforcement learning is concerned with how a software agent learns to act in a particular environment to accomplish a certain goal, such as the maximization of a particular kind of reward. As a result, it is well-suited to resolving control difficulties, such as those seen in Radio Resource Manager. Following that, we look at a model-free case in which the issue is fully described with three components: the state, action, and reward. The state *s* is a tuple of variables referred to as features that uniquely describe the agent’s surroundings in terms of the task at hand. The action *a* denotes the agent’s modification of the surrounding environment. The reward function *r* is a multi-objective scalar function that quantitatively represents the agent’s goal. The agent’s interaction with the environment throughout time is characterized by a sequence of tuples (*s*_t_, *a*_t_, *r*_t+1_, *s*_t+1_), each of which represents a state transition caused by the agent executing actions on the environment and receiving rewards. The goal of reinforcement learning is to construct a policy from a collection of transitions that, given a state, generates the action to perform to maximize the cumulative long-term reward. Thus, a reinforcement learning system establishes a connection between rewards and distantly related behaviors—the technical word for this is credit assignment. A reinforcement learning approach should quickly transition an agent from a blank slate state, in which it has no idea of how to behave, to an ideal state. Making the fewest potential mistakes on the way to quasi-optimal behavior is referred to as regret minimization, a notion that is strongly connected to the problem of balancing environmental exploration and knowledge extraction. This progression from exploration to exploitation may take a number of forms.

### 4.6. Blockchain-Enabled Network Slicing Agents (BENS-Agents)—An Intelligent Multi Agent System

**Figure 4:**
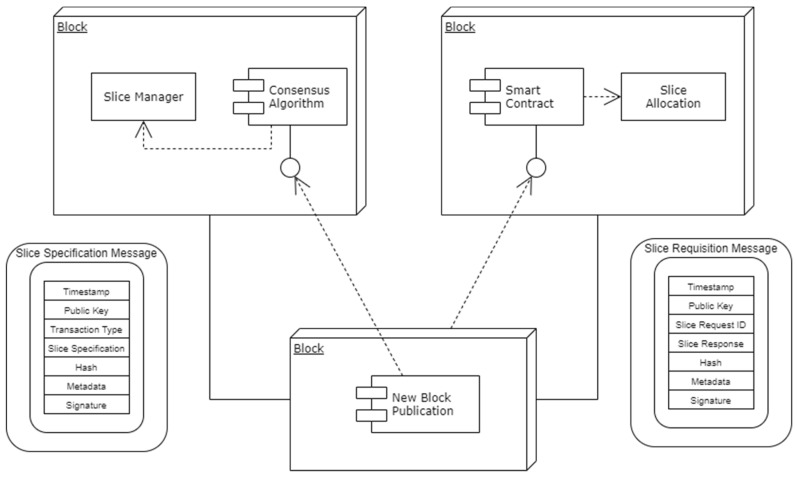
BENS-B5G model.

For the sake of the design, a private blockchain has been used as shown in Figure 4. Employing the user front end, consent is written or recorded on the network of blockchains that are based on the Hyperledger Fabric after signing up for the service and saving the appropriate information on the database. The Core Network Slice ID, the Access Network Slice ID, and the Network Access Slice Information are all included in the details of the consent. A user has the option of mentioning whether they are ready to grant entire or partial access to the resource. When a user records their consent data on blockchain, an administrator has the ability to verify those details and then provide access to research organizations. Using the front end of the organization, the organization on the network is able to view the consent information of the user, and access may be sought from the healthcare administrator if the user has provided full access. The organization’s front-end interface may also be used to request more access if necessary. When a research organization asks for further consent from a user, the user’s account will be updated with a notice. The information will be logged in the network as a transaction regardless of the user’s decision on whether or not to comply with the request. In the event that the request has been granted, the administrator will have permission to exchange the data with the organization on an as-requested basis. It is possible for the network to keep a record of the specifics of an individual user’s request to have their data removed. The transaction will be updated with the new information. After verifying the information provided by the user, the administrator promptly removes the information from the database.

An Intelligent Agent (IA) adapts the AI properties, such as:

- Actions—the responding capability of an agent towards environmental changes and events;
- Perceptions—environment-provided data, which on further process, the data will be accumulated as information;
- Goals—system objectives;
- Events—update beliefs and perform actions;
- Beliefs—the processing of environment-provided accumulated information;
- Messages—the interaction of agents;
- Plans—achieving goals and handling events;
- Protocols—interaction rule sets.

#### 4.6.1. BENS Agent Blocks

Figure 4 represents the blockchain consortium which depicts the slice requirements and requisition message formats. In addition to the vast amount of user equipment competing to access a large number of spectra, various applications will have their own QoS requirements. The QoS requirements are to be considered for channel assignment. The QoS-aware Application-specific agent behavioral model is formulated in such a way that each device enabled with Intelligent Agents will intelligently make spectrum access decisions with the help of their observations. QoS requirements are incorporated into the learning process, and the learning process is enhanced with learning transfer and cooperative learning mechanisms to adapt to the distributed heterogeneous environment. When new user equipment joins the environment, or when it applies for a new service, it can directly search for the expert agent among the neighbors, and by utilizing the learning transfer model, it can infer the knowledge about the data it seeks from the expert agent. The actions and events are in the cooperative learning model, and the data shared can be utilized for decision making.

BENS incorporates Neural Networks to switch from the observed state to actions through various layers, instead of relying on Q-values and its memory storage. Any high-scale complex model can be realized using Neural Networks with the help of multi-dimensional data. Additionally, by using the experience replay and generalization capabilities of Neural Networks, BENS may enhance network performance. A large number of communication entities can attempt to access a highly constrained spectrum resource in 5G and beyond 5G networks. This can be framed as a learning problem of the Multi Agent System. Every agent expects the network environment to offer spectrum resources and allied services by being in a state in which they can observe the environment. Our proposed approach to design a multi-agent system comprises of two stages, as follows:

#### 4.6.2. Training Stage

The training stage for a BENS multi-agent system is proposed to have the following list of behavioral characteristics

- The agents are all the communication linkages, and the wireless network serves as the environment.
- By integrating with the environment, each agent intelligently monitors its present condition.
- Then, based on the learned policy, it makes a decision, and selects an action.
- Following that, the environment provides each agent with a new state and an instant reward.
- All agents intelligently learn new policies in the future time step based on the input.
- An established replaying method is used to increase the rate of learning, the efficiency of learning, and the stability of learning toward the ideal policy for expansive access control.
- The storage memory is used to store the training data.

#### 4.6.3. BENS Training Stage Algorithm (BENS-T)

The BENS-T Algorithm [Algorithm 1] is a reinforcement learning algorithm that operates inside a 5G environment. The algorithm is based on a Markov model that has numerous states and actions. The algorithm decides the action to take in each state by accumulating knowledge about the values associated with each possible action. In addition to that, it combines many exploration methodologies within its overall architecture. It has been shown that the algorithm will eventually arrive at the best course of action for each state.

**Algorithm 1.** BENS—Training Algorithm (BENS-T).
**Input**: BENS framework, scenario emulator, and all applications’ QoS requirements.**For** every session *i* = (1, 2, …, to M)^instancs^, perform the following: **Initialize**: every agent Q-network where, function *Q*(*p*, *b*), rule-based approach *φ*(*p*, *b*), load β, refactor *W*; **Perform** process *Instance* = (0, 1, 2, to *F*); Every agent keeps track of its condition *T_s_*; Randomly **select** action A_c_ with probability λ; Alternatively, randomly select action A_c_ with probability = max arg a∈A *Q_t_*(*P_s_*, *b_s_*, *µ_s_*); Carry out an **action** at, then collect a reward *W_ins_*; **Record** a fresh state *T_s_*_+1_; **Store** refactor *W_ins_* = (*p_s_*, *b_s_*, *W*(*p_s_*, *b_s_*), *T*_*s*+1_) in memory **O**; **Repeat** **for** every agent, proceed;  Randomly sample a micro-data *k_s_* in **O**;  **Add** µ_s_—Update;  Incline to update µ_s+1_;  **Update** φ, the policy with Q-max_value;  **Perform** action on φ; **Conclude** the loop for;**Conclude** the **loop for;**
Return: Return trained BENS models.


#### 4.6.4. BENS-Agent Learning Algorithm (BENS-AL)

Algorithm 2 aggregates and groups agent data by observing and identifying the neighborhood agents. The agents are performed with actions and rewards with the decisions based on aggregated data.

**Algorithm 2.** BENS-Agent Learning Algorithm (BENS-AL).
**Input:** BENS structure, simulation of environment parameters, and QoS parameters of end applications.**Initialize:** Every agent in observation stage *S*;Learning transfer: If found new agent/service;**Identify** the neighbors of agent and transfer information; **Analyze If** the information exist Identify Expert Agent *EA*; **Extract** *EA* information; Check and **do** database update *D_t_*; Set transmission rate µ; **Select** an appropriate action *A_t_*; Cooperative learning: Every group *CG* share actions and observed data; Aggregate the group data *CG_D_*; Set join policy *π_g_*(*CG_g_*) and aggregated value *A_g_*(*CG_g_*, *b_g_*), and Set aggregated action *AG_g_*; Execute action *AG_g_*, receive reward *RW_g_*; **end if.**
**End BENS-AL**



## 5. BENS-5G: Procedure for RAN Slicing in 5G-CORE

We devised a mathematical model of the CRN-enabled 5G in different scenarios:

1. No channels are reserved;
2. Certain idle channels are allocated for the PUs. To begin, a wireless channel is mathematically designed for NONRES and RES.

Whenever a wireless channel is devised, it is always as a two-state Markov process. An Occupied State indicates that a PU is now occupying the current channel. An Unoccupied State is a channel that is not in use by a PU, and is thereby accessible to an SU. Allow for M wireless channels in the system. As a result, the transition probability *P*(*a*,*b*) represented in Equation (1) indicates that there are a number of vacant channels in the present frame, and b in the following frame.

(1) $$P_{a,b}=\sum_{y^{\prime }=\max\left(a-b,o\right)}^{\min\left(a,a-b\right)}\left(\begin{array}{c} a\\ y’ \end{array}\right)\;P_{00}^{a-y’}\;P_{01}^{y’}\;\left(\begin{array}{c} A-a\\ x’ \end{array}\right)\;P_{11}^{a-y’}\;P_{10}^{y’}$$

where, in this case, *x* = *b* − *a* + *y*’. The *y*’ and *x*’ represent the number of channels that have changed their state from Vacant to Occupied and Occupied to Vacant, respectively. Spectrum sensing with inaccuracies introduces malfunction and misses detection issues. As a result, the number of observed vacant channels is a measure of the total range of actual vacant channels, indicated by Equation (2): *a*′(*a*), which is given by:

(2) $$a^{\prime }\left(a\right)=a-a\left(p_{fa}\right)+\left(A-a\right)\left(1-p_{md}\right)$$

where $p_{fa}$ is the false alarm probability;

$p_{md}$ is the detection probability;

1 − $p_{md}$ is the miss-detection probability.

### 5.1. Assumptions

There are N channels, each of which is shared by primary and secondary users, with the main user taking precedence over the secondary user(s). Calls from primary users occur at a rate of λ1, while calls from secondary users occur at a rate of λ2. The µ1 and µ2 are the respective service rates. The following assumptions are made:

(1) When a PU joins a channel already occupied by SUs, the SU is constantly aware of the PU’s presence. Notably, this causes a temporary clash with the principal user. A search for a new channel is then initiated by the SU. The SU will check the remaining channels at random until it finds a free one or determines that all the bands are occupied. The likelihood of a free channel being occupied is dictated by the probability of a false alarm occurring, and the probability of an occupied channel being free is given by the probability of a miss detection occurring.
(2) All state changes are immediate, implying that the time required to find a free channel is insignificant.
(3) An SU is aware of the channels that are being used by other SUs and will avoid them. For example, the relevant information may be conveyed through a signaling channel.
(4) A PU is aware of the channels that other PUs are using, ensuring that there are no clashes between primary users.
(5) In the event of a channel collision (between the PU and SU), both conflicting users continue the transmission in the channel. Notably, the collision between the PU and SU is believed to be brief, and does not result in the PU exiting the channel.
(6) The sequence in which new free channels are identified is random. When a free channel is found, or all channels are confirmed to be occupied, the search comes to an end. Many channels may be searched before halting the entire procedure.

### 5.2. Procedure Model

A two-dimensional Markov chain is used to represent the system. Tuples *P* and *Q* are used to represent the number of channels used by primary and secondary users, respectively, in the system states. There are two primary user and one SU in the (2,1) example. Assume that K is the number of channels in the system that are accessible. N is the maximum number of users, primary and secondary, on a given channel. As a result, the limitations being enforced are 0 ≤ *P* ≤ *K*, 0 ≤ *Q* ≤ *K* and 0 ≤ *P* + *Q* ≤ *K*. Let *z*(*P*, *Q*) be the duration spent in state by the system state (*P*, *Q*), which signifies the probability of steady-state.

### 5.3. State Transitions

**Figure 5:**
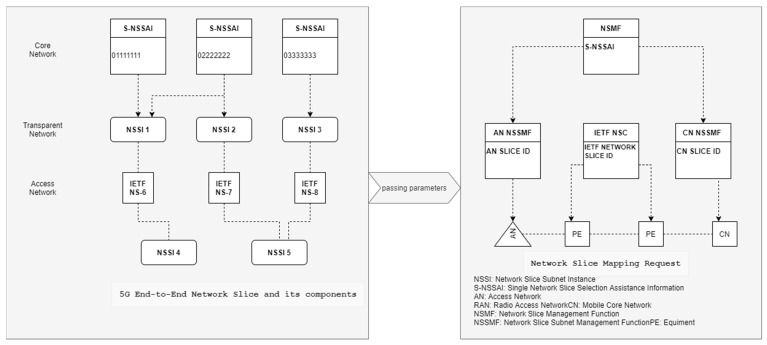
Network slice mapping structure for 5G.

The following chart depicts the state transition diagram where three channels are used. Iterating through all possible channel and detection event sequences yielded the state-dependent transition rates. Consider the progression from state (1,1) to state (2,1). (1,0). The detection events and the channel search order changes are shown in this section. One of two occurrences might cause this transition to occur. During the first scenario, the current secondary user call is terminated at a rate of two, which reduces the number of secondary users by one. When a new primary user joins an existing secondary user’s channel, it forces the secondary user to hunt for new channels. A collision between an existing secondary user and an already existing PU results in both calls being dropped, leaving just the new primary (1,0). To go a little deeper, start with the current state of affairs (1,1). It is possible for the new PU to utilize either an existing channel, or one that is currently being used by a secondary user, since the main user is aware of any existing PU. Since the new primary user is not worried about secondary users, they will approach the primary user’s channel with a probability of 0.5. This results in a collision with probability P_M_ when the secondary user connects straight to the current user’s channel with a probability of 0.5. Secondary users may connect to a free channel (with a chance of 0.5) first, and then continue to the primary user channel, and obtain a misdetection if they are unsuccessful. The process of movement is from a state (2,1) to another (3,0). The secondary user will always be forced by the new primary user to leave the channel, and search for other accessible channels. It is possible for the secondary user to accurately identify that the primary user is consuming the two extra channels with probability P. Figure 5 represents the state transitions which happen in 5G CORE functions during slice mapping.

### 5.4. BENS Algorithm for Channel Reservation (BENS-R)

The BENS-R algorithm [Algorithm 3] optimises channel usage by implementing an adaptive channel reservation method based on PU activity. It also offers priority-based access to various kinds of SU traffic for which the sample given in Table 1, which leads to an increase in network system capacity and heterogeneity.

**Algorithm 3.** BENS Algorithm For Reservation (BENS-R).**Input**: N number of available channels in the CRN,
   N_np_ Number of PU-occupied channels in Unreserved CRN
   N_ns_ Number of SU-occupied channels in Unreserved CRN
   N_rp_ Number of PU-occupied channels in Reserved CRN
   N_rs_ Number of SU-occupied channels in Reserved CRN
   f_a_ number of failed channels in the CRN
   max_res_ number of maximum reserved channels
   values K_i_ such that K_a+1_ < K_a_ < K_a − 1_ < K_a − 2_...< K_i_ <...<K_0_;
   i = 1 to a, where K_a_ and K_a+1_ = 0
**Output**: M number of reserved channels in the CRN, 
**Step 1: Calculate** β = (p_n_ + q_n_ + p_r_ + q_r_)/(Q − e)
**Step 2: Find** M_Available_ = Q − (p_n_ + q_n_ + p_r_ + q_r_ + e)
**Step 3: Find** R_N_ = p_r_ + q_r_
**Step 4: while**(k > 0) do 
   if (k_i + 1_ ≤ β < k_i_) 
   thentraffic_threshold_level = j;
   k = k − 1; repeat
**Step 5: do** break;
**Step 6: end**
**Step 7:** Switch to operating mode:
Operating mode = 0 indicates that the objective is to increase the retainability of current monitoring services.Operating mode = 1 denotes the increase in the availability of channels for fresh users.
**Step 8: if** selected Operating mode = 0 then
**Step 9:** M’ = max_res_ − traffic_threshold_level
**Step10: end**
**Step 11: if** selected Operating mode = 1 then
**Step 12:** M’ = max_res_ − (a-traffic_threshold_level)
**Step 13: end**

## 6. Performance Analysis

We analyzed the benefits of the reservation of channels strategy in the CRN for individual SUs and the entire system. To begin, the analytical models developed in previous sections are utilized to analyze the traffic for each SU. Additionally, a simulation model for SU traffic-based CRNs is developed in OMNET++ to investigate and confirm the performance increase in the Reservation-enabled CRNs, compared to CRNs with unreserved channels.

**Figure 6:**
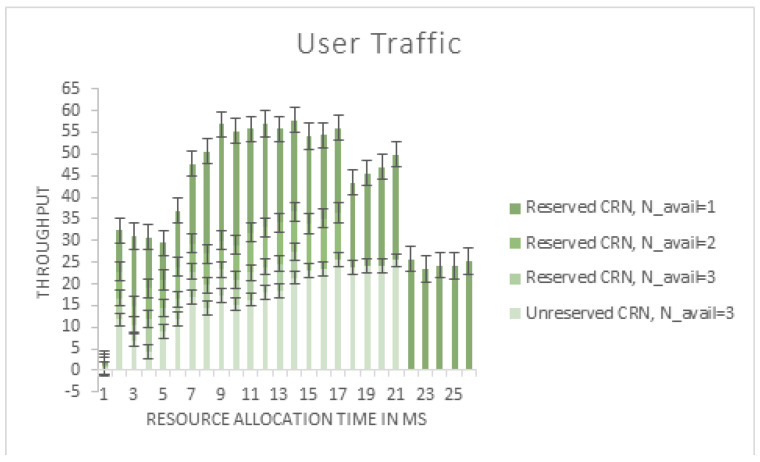
Variation in user traffic with respect to channel reservation time (slice request).

From Figure 6, as the channel reservation reduces the Secondary User’s spectrum use, it is evident from the figure that the reserved CRN has a lower Secondary User throughput than the unreserved CRN. As the number of reserved channels for the Pus decreases, the number of Sus allowed into the CRN increases, resulting in an increase in the overall system throughput, as depicted. However, this increases the amount of interference and the number of incorrect packets received by the Secondary Users.

**Figure 7:**
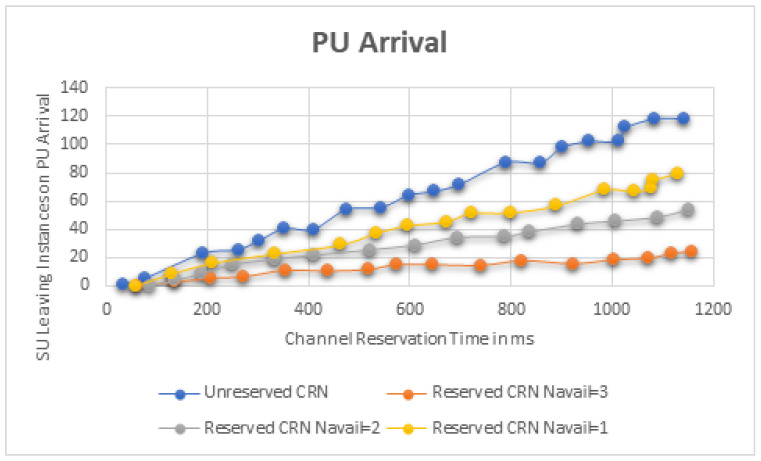
PU Channel Switching.

Figure 7 depicts a temporal average plot of the proportion of time during which incorrect packets are received by receivers at the SUs’ end. It is observed that in an unreserved CRN, incorrect packets account for about 4/5th of the entire transmission time, but in a reserved CRN, this proportion is drastically decreased. The overall number of SUs that drop likewise reduces significantly in the reserved CRN. The analytical model for SU traffic demonstrates the user-centric requirement of a channel reservation system for ensuring QoS in real-time applications. On the other hand, the simulation model demonstrates the reserved CRN’s benefits as a system, and further verifies the reserved channels for the PUs. Thus, the contribution of this study to a comprehensive performance assessment has been effectively demonstrated.

### 6.1. Simulation Results and Inferences for BENS-R

Table 1 depicts the simulation parameters being configured in Omnet++ to analyze the performance of network convergence of our proposed BENS-5G model multi-agent system with distributed and centralised multi-agent system

**Figure 8:**
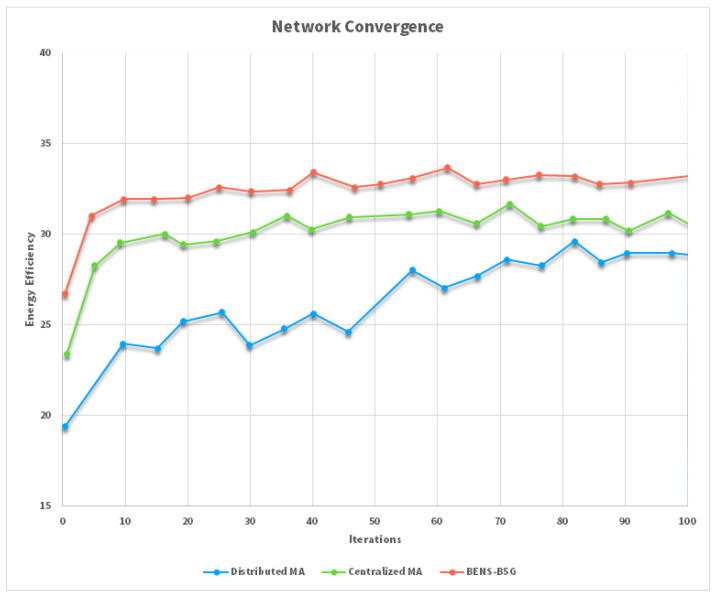
Convergence comparison.

We have compared the proposed Blockchain-Enabled Network Slicing (dubbed BENS, which incorporates both transfer and cooperative learning mechanisms) to the fully distributed multi-agent RL-based massive access approach, in which each communication link determines its sub-channel assignment and transmission power strategy independently of other communication links. As seen in the Figure 8, the suggested learning technique greatly outperforms the fully distributed learning and centralized MA approaches in terms of EE performance and transmission success probability. The suggested technique achieves a quicker convergence rate via the use of transfer learning and cooperative learning mechanisms to boost the learning efficiency and speed. Since it does not need device cooperation, the completely dispersed strategy is straightforward; however, it results in a poor global performance, which results in a low EE value, and a low likelihood of transmission success. One of the most important contributions made by the algorithms developed for this work is the efficient management of network resources, with a particular emphasis on the computational and networking resources of core networks. In 5G networks, however, computing resources have been placed closer to the end users via edge-computing technologies in order to reduce excessive end-to-end latency. As a result, the problem of resource allocation will become much more difficult, particularly for online conditions. This is due to the fact that these conditions will require more computational time in order to more accurately categorize the best resources and paths when it comes to allocating network services that are sensitive to delay. Additionally, in migration situations, a shorter migration time is an essential key performance indicator regarding the effectiveness of performing resource allocations for moving services in preparation for a real-time scenario.

### 6.2. Inference

As mentioned earlier, most research on dynamic spectrum allocation in CRNs is focused on spectrum sensing and allocation. However, minimal research on dynamic spectrum access with channel reservation and the mobility of the CRNs is based on channel reservation. Very few studies have examined the use of PU and SU dynamics in CRNs to enhance hand-off and mobility management in typical cellular networks. We allow for a change of the PU—SU characteristics to satisfy the needs of new services. These modifications are accomplished via the use of consensus and BENS algorithms that regulate PUs and SUs through negotiation and allocation procedures. As a result of the discussion above, we can affirm that the Blockchain Consensus Spectrum Management algorithm and BENS Model used to control RAN in 5G is capable of providing very efficient solutions to a variety of cognitive radio difficulties. The use of the collaborated framework in 5G−CORE is intriguing, and represents unexplored research. 

## 7. Conclusions

In a 5G−CORE network, we presented a system for dynamic spectrum sharing, and concentrated on the setup of RAN Slicing in 5G−CORE using a consensus algorithm. We also presented a learning strategy for the extension of spectrum sharing in 5G−CORE to overcome the dynamic spectrum access issue in 5G. Our proposed BENS approach achieves higher energy-efficient performance and greater probability-of-success rate for transmission than other distributed and centralized learning approaches. Our proposed approach proves to have a good and faster convergence speed by assuming learning handover and combining collaborative techniques for learning. Furthermore, we discussed the issues of interference and power allocation and proposed the methods for solving the issues. The proposed methods are feasible for implementation in any vendor-specific 5G−CORE architecture. The Dynamic Spectrum Allocation Framework for the 5G environment did not consider the applications that operate on 5G radio environmental dynamics. We suggest that future research directions must be to identify the heterogenous applications and their traffic over 5G and beyond networks, to enhance the proposed work.

## Figures and Tables

**Table 1:** Simulation Parameters.

Parameters	Values
Cell Radius	500 m
Carrier Spectrum/Bandwidth	2 GHz, 100 MHz
Inter-device Distance (max.,)	75 m
Number of Primary devices	100, (200–600)
Number of channels	100
Number of SU devices	500, (1000−3000)
Latency benchmark value	(1, 2, 4, 6, 8)
SINR Threshold	5 dB
Max Transmit power of each device	500 mW
Device power consumption	50 mW
Device power consumption	50 mW
Packet size	1024 bytes

## Data Availability

The data used to support the findings of this study are available from the author (postman3@dongguk.edu) upon request.
